# Preoperatively undiagnosed papillary thyroid carcinoma in patients thyroidectomized for benign multinodular goiter

**DOI:** 10.20945/2359-3997000000017

**Published:** 2018-03-23

**Authors:** Fausto Fama, Alessandro Sindoni, Marco Cicciu, Francesca Polito, Arnaud Piquard, Olivier Saint-Marc, Maria Gioffre´-Florio, Salvatore Benvenga

**Affiliations:** 1 University Hospital of Messina Department of Human Pathology in Adulthood and Childhood “G. Barresi” Messina Italy Department of Human Pathology in Adulthood and Childhood “G. Barresi”, University Hospital of Messina, Messina, Italy; 2 University Hospital of Messina Department of Biomedical and Dental Sciences and of Morphological and Functional Images Messina Italy Department of Biomedical and Dental Sciences and of Morphological and Functional Images, University Hospital of Messina, Messina, Italy; 3 University Hospital of Messina Department of Clinical & Experimental Medicine Messina Italy Department of Clinical & Experimental Medicine, University Hospital of Messina, Messina, Italy; 4 Regional Hospital of Orleans Department of General, Endocrine and Thoracic Surgery Orléans France Department of General, Endocrine and Thoracic Surgery, Regional Hospital of Orleans, Orléans, France; 5 University Hospital of Messina Messina Italy Master Program on Childhood, Adolescent and Women's Endocrine Health, University Hospital of Messina, Messina, Italy; 6 University Hospital of Messina Messina Italy Interdepartmental Program on Molecular & Clinical Endocrinology, and Women's Endocrine Health, University Hospital of Messina, Messina, Italy

**Keywords:** Incidental thyroid cancer, benign thyroid disease, multinodular goiter, total thyroidectomy, papillary thyroid cancer

## Abstract

**Objective:**

Incidental thyroid cancers (ITCs) are often microcarcinomas; among them, the most frequent histotype is the papillary one. The purpose of this study was to evaluate the rate of papillary thyroid cancer (PTC) in patients thyroidectomized for benign multinodular goiter.

**Subject and methods:**

We retrospectively evaluated the histological incidence of PTC in 207 consecutive patients who, in a 1-year period, underwent thyroidectomy for benign multinodular goiter. All patients came from an iodine-deficient area (Orleans, France) with three nuclear power stations located in the neighboring areas of the county town.

**Results:**

Overall, 25 thyroids (12.1%) harbored 37 PTC, of which 31 were microcarcinomas. In these 25 PTC patients, mean age was 55 ± 10 years (range 30-75), female:male ratio 20:5 (4:1). In 10 patients (40% of 25 and 4.8% of 207), PTCs were bilateral, and in 7 (2 with microPTCs) the thyroid capsule was infiltrated. These 7 patients underwent central and lateral cervical lymph node dissections, which revealed lymph node metastases in one and two cases, respectively. Radioiodine treatment was performed in 7 cases. Neither mortality nor transient and permanent nerve injuries were observed. Four (16%) transient hypocalcaemias occurred as early complications. At last follow-up visit (mean length of follow-up 17.2 ± 3.4 months), all patients were doing well and free of any clinical local recurrence or distant metastases.

**Conclusion:**

With a 12% risk that multinodular goiter harbors preoperatively unsuspected PTCs, which can have already infiltrated the capsule and that can be accompanied by PTC foci contralaterally, an adequate surgical approach has to be considered.

## INTRODUCTION

In the thyroid literature the term *incidental* has been used to indicate an unsuspected finding; nevertheless, the nature of the incidental finding depends on the clinical context in which the nodules are found. Considering thyroid gland, the identification of thyroid cancer may be classified into 3 broad categories: 1) clinically detected cancer (not incidentally detected), 2) radiologically detected cancer (clinically unsuspected), and 3) pathologically detected cancer (clinically and radiologically unsuspected) (1). Incidental thyroid cancers (ITCs) are often microcarcinomas, most frequently of the papillary histotype (2–6); the mean tumor size of ITCs decreased during the last decades (3,6). Namely, Boucek and cols. (7) divided ITC diagnoses into four different categories: i) neoplasms found incidentally after thyroidectomy whereas preoperatively only benign pathology was known; ii) neoplasms that were diagnosed incidentally on imaging, mainly ultrasonography (US), and that were evaluated further and confirmed by fine-needle aspiration cytology (FNAC); iii) neoplasms that appeared clinically as lymph node metastases, with primary thyroid carcinoma detected only at histological specimen examination; iv) thyroid cancer that is localized in ectopic thyroid tissue with clinical symptoms or metastases present. Besides these four groups, Liu and cols. (8) proposed another ITC group including patients that presented, despite benign thyroid disease ascertained at imaging and definitive histology, regional or distant lymph node metastases from primary thyroid carcinoma not identified at thyroid pathological examination.

An ITC discovered at histology, after surgical removal of the thyroid for a benign pathology, is the most frequent event (9–12). In thyroidectomy specimens, ITC prevalence ranges up to 40% (2). In autopsy studies, the reported prevalence of ITC ranges from 0.01% in USA to 35.6% in Finland (7). Recently, a study from U.S.A. have documented that most counties with the highest thyroid cancer incidence are in a contiguous area of eastern Pennsylvania, New Jersey, and southern New York State; radioactive exposures from 16 nuclear power reactors within a 90-mile radius in this area have indicated that these emissions are a likely etiological factor in rising thyroid cancer incidence rates (13).

Over the last 30 years, there has been an increase in the overall incidence of thyroid cancer, from 3.6 (in 1973) to 8.7 (in 2002) per 100,000 inhabitants (14). The incidence rate of papillary thyroid cancer PTC rose up more than any other malignancy (15,16), up to 93% of all thyroid cancers in Japan and up to 85.3% in Western countries (7). PTC is the most common histotype and microPTC represents up to 30% of all forms of papillary cancer (17).

The very recently released American Thyroid Association guidelines on thyroid nodules and cancer underscore that “a recent population based study from Olmsted County reported the doubling of thyroid cancer incidence from 2000-2012 compared to the prior decade as entirely attributable to clinically occult cancers detected incidentally on imaging or pathology” (18–20). By 2019, one study predicts that papillary thyroid cancer (PTC) will become the third most common cancer in women (21).

The purpose of this study was to evaluate the rate of histologically detected PTC in consecutive patients who were thyroidectomized for benign multinodular goiter (MNG) throughout a 1-year period at a single endocrine surgery unit. Of note, this surgery unit and patients’ residence is located near to three nuclear power units. Our data were compared with those of the English language literature on the ITCs.

## SUBJECTS AND METHODS

All patients of this retrospective cohort were admitted on the same day of the surgical procedures, performed by 3 experienced endocrine surgeons under general anesthesia. Preoperatively, patients were studied by means of neck US and routine blood test, including hormones levels. The American Society of Anesthesiologists (ASA) physical status was assessed in all patients. In order to obtain a more homogeneous cohort of patients, we excluded patients with suspicious characteristics of the thyroid nodule(s) (i.e. irregular margin and/or contour and/or shape, calcifications, hypoechogenicity, vascularity or local invasion/lymph node metastases) at US (n = 19), history of previous neck surgery (n = 7), history of malignancy in other organs (n = 5) and ASA score greater than 4 (n = 2).

Parathyroid glands and recurrent nerves were identified in all cases, and specimens sent to pathologists for the frozen section; no cervical drains were placed systematically. Patients were discharged, generally in the second post-operative day, with a prescription of a weight-adjusted thyroxine treatment. Patients were referred to our endocrinological outpatient surveillance program. We defined microcarcinoma or macrocarcinoma any cancerous nodule up to 10 mm or greater than in maximum diameter, respectively. When multifocality occurred, we considered the largest neoplasm and classified according to its anatomical site. For purpose of comparison with the international literature, we run a PubMed search entering the words “incidental thyroid cancer” or “incidental thyroid carcinoma”. The search was updated until November 2016. The search was limited taking into consideration only original papers. The references of the retrieved articles were also checked so as not to miss important clinical studies. Original articles reporting data about patients who underwent surgery for suspicious or preoperatively documented disease, as well as editorials, commentaries, review articles and similar types of articles were excluded. Animal studies were also excluded. Two researchers (A.S., S.B.) independently reviewed the titles and disagreements were resolved in a consensus meeting.

### Statistical analysis

Results are expressed as mean ± standard deviation (SD). Laboratory data without normal distribution were described using median and percentile values. Fisher's exact test was used to analyze categorical data. The level for statistical significance was set at P < 0.05. Statistical analysis was performed using Kyplot v2.0 beta 13 version.

## RESULTS

**Figure 1:**
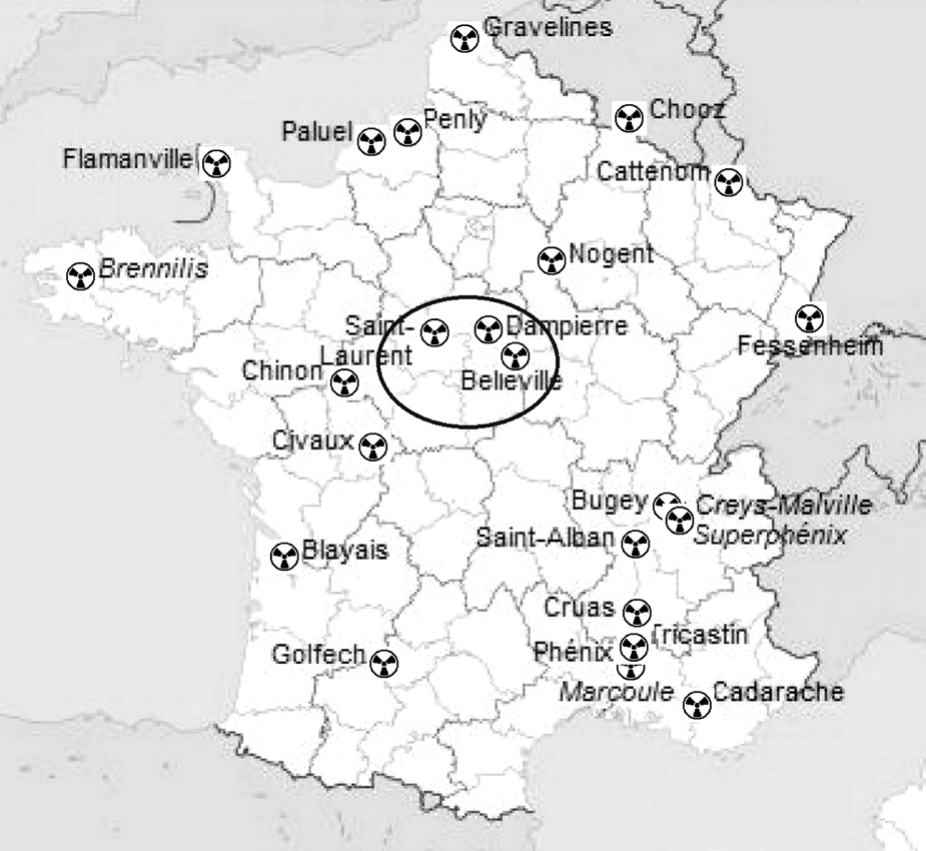
Topography of nuclear power plants in the neighboring areas of Orleans, France (ring).

In our study from a French endocrine surgery unit, we retrospectively reviewed 207 consecutive patients, 169 were females (mean age of 53.0 ± 12.6 years [range 18–79]) and 38 males (mean age of 54.9 ± 14.2 years [range 21–78]), who underwent total thyroidectomy (TT) for benign bilateral MNG from January to December 2014. All patients came from an iodinedeficient area (Orleans, France) (22) with three nuclear power stations located in the neighboring areas of the county town (Figure 1). Clinico-laboratory data of all patients are shown in Table 1.

**Table 1:** Demographic and clinico-laboratory characteristics of patients undergoing total thyroidectomy for benign multinodular goiter

	All patients (n = 207)
Age, years mean ± SD (range)	53.9 ± 13.9 (18 – 79)
TSH, uUI/ml median (interquartile range)	1.54 (1.07 – 2.27)
FT3, pmol/L median (interquartile range)	5.6 (4.3 – 6.2)
FT4, pmol/L median (interquartile range)	15.2 (12.8 – 16.9)
Tg, ng/ml median (interquartile range)	24.2 (19.5 – 35.7)

SD: standard deviation; Tg: thyroglobulin.

Over the 12-month chronological window of our study, in 25/207 patients (12.1%) we discovered 37 preoperatively unsuspected, and therefore ITCs, all being PTCs. Their ASA score of these patients was ASA1 (n = 4), ASA2 (n = 18) and ASA3 (n = 3). Mean hospital stay was 1.1 ± 0.3 days; 23 (92%) were discharged on the 1^st^ post-operative day and 2 on the 2^nd^ post-operative day.

Of these 37 PTCs, 31 (86.1%) were microPTCs, with a maximum diameter ranging 1 to 6 mm, while 6 were macroPTCs (diameter range 12-16 mm). Overall, mean age of the 25 patients was 55 ± 10 years (range 30-75) with 20 being females (F:M ratio = 4:1). Patients with macroPTCs were 7 years older than patients with microPTCs (Table 2). Histopathological examination showed bilateral MNG in all cases (mean weight of the thyroid glands: 53.6 ± 45.7 g) and the additional presence of chronic lymphocytic thyroiditis or Hashimoto's thyroiditis (HT) in 6/25 patients (24%, all with positive thyroid peroxidase and thyroglobulin autoantibodies). Thyroid tumors were monofocal in 15 patients (all microPTCs; 15/37 tumors in 15 patients) and multifocal in 10. Of these multifocal PTCs, 16 were microcarcinomas and 6 macrocarciomas. In 5 of the 10 patients microPTCs and macroPTC coexisted.

**Table 2:** PTC patients and tumours characteristics

	Right lobe	Left lobe	Isthmus	Total
No. of nodules	17	16	4	37
(%)	(46.0%)	(43.2%)	(10.8%)	(100%)
No. of patients with multifocality	4	5	1	10/25
(%)	(16%)	(20%)	(4%)	(40.0%)
**microPTC**				
No. of nodules	14	14	3	31
(%)	(45.2%)	(45.2%)	(9.6%)	
Mean diameter (mm) ± SD	4.4 ± 2.7	3.9 ± 2.5	1	3.8 ± 2.6
Mean age (years) ± SD	55.9 ± 10.2	52.2 ± 7.7	57.0 ± 18.7	54.4 ± 9.9
F:M ratio	3.7:1	3.7:1	3:0	4.2:1
**macroPTC**				
No. of nodules	3	2	1	6
(%)	(50%)	(33.3%)	(16.7%)	
Mean diameter (mm) ± SD	13.0 ± 1.7	12.5 ± 0.7	30	15.7 ± 7.1
Mean age (years) ± SD	60.0 ± 13.5	67.5 ± 9.2	52	61.2 ± 11.1
F:M ratio	3:0	2:0	0:1	5:1

PTC: papillary thyroid carcinoma; SD: standard deviation; F: female; M, male.

Multifocal PTCs, including coexistence of micro (n = 16) and macroPTCs (n = 6), were always bilateral. Of the 15 monofocal microPTCs, 8 were right-sided, and 7 left-sided (Table 1). MicroPTCs and macroPTCs did not differ in distribution if we considered the right lobe-left lobe-isthmus location (P = 0.836 by Fisher's exact test) or the classification among the upper-middle-lower-isthmic localization in the thyroid (P = 0.334 by Fisher's exact test). Of the 6/207 patients with HT, 2/6 (33.3%) had 4 of the 37 PTCs, all 4 tumors being microPTCs.

Seven supplementary central and lateral cervical lymph node dissections were carried out, because 2 microPTCs and 5 PTCs were infiltrating the thyroid capsule at frozen sections. Lymph node metastases were found in one and two patients, respectively. Radioiodine treatment, with a dose of 100 mCi, was performed in 7 cases, because of the presence of poor prognostic factors such as capsular infiltration, macroPTC and/or multifocality.

Neither mortality nor transient and permanent nerve injuries were observed. Four (16%) transient hypocalcaemias occurred as early complications, and were successfully treated by a 6-week combined cholecalciferol and oral calcium supplementation.

At last follow-up visit (mean length of follow-up 17.2 ± 3.4 months), all patients were doing well and free of any clinical local recurrence or distant metastases.

An overview of the literature is summarized in Table 3 (23–68). Reported prevalence of ITC at surgery ranges between 2% and 40% (1,2,17,23–68): in Europe it varies from 2.2% to 27.4% and in the United States it varies from 3.3% to 33%. In some European countries, such as Romania, Czech Republic, Ukraine and Poland, the frequency of thyroid cancer showed a lower range (i.e. from 5 to 9.2%); in Turkey, excluding the study from Tasova and cols. (46), there has been a lower variation range in its reported incidence (7-10%). Rates from other European countries were: 12.5 % from Belgium, 10.4-11.1% from Italy and 12.0% from Greece.

**Table 3:** Summary of the literature on thyroid cancers that were discovered incidentally at thyroidectomy in patients underwent surgery for benign thyroid disease

Author (ref)	Years of study	Country	Patients studied	Surgical procedure	Rate of cancer	Comment
Fama’ and cols., this study	One (2014)	France	207 pts	TT	In 25/207 (12.1%) pts, 37 PTC were detected (31 microPTCs and 6 PTCs)	10/25 (40.0%) pts had multifocal tumours; all pts underwent surgery for MNG
Daumerie and cols., 1998	1976 - 1995	Belgique	93 pts	TT in 16/47 (34.0%) pts with MNG (group I), PT in 39/46 (84.8%) pts with a solitary hot nodule (group II)	2/16 (12.5%) pts (group I) and 5/39 (12.8%) pts (group II) had microTC, with a total prevalence of 12.7%	
Dănilă and cols., 2008	2000 - 2006	Germany	92 pts	TT	2/92 (2.2%) pts had microPTC (tumour size ranged from 3 to 5 mm)	All pts had GD; multifocality and lymph node involvement were not detected
Pezzolla and cols., 2014	Jan 2010 - Jun 2013	Italy	256 pts	Surgical procedures in tumours pts 28/256): TT in 27/28, PT in 1/28 PT	In 28/256 (10.9%) pts, 40 TCs were detected (29 FV-PTC, 10 PTC and 1 FTC)	Pts underwent surgery for: 176 pts (MNG), 67 pts (GD), 12 pts (UNG) and 1 pt (PD)
Pezzolla and cols., 2010	n/a	Italy	165 pts	n/a	30/165 (18.2%) pts had TC (18 PTC, 6 FTC, 5 FV-PTC and 1 oncocytic carcinoma); 15/30 (50%) were microcarcinomas	Pts underwent surgery for: 132 pts (MNG), 30 pts (UNG), 2 pts (PD) and 1 pt (GD)
Costamagna and cols., 2013	2001 - 2009	Italy	568 pts	TT in 499/568, PT in 69/568	53/568 (9.3%) pts had TC (24 FV-PTC, 20 PTC, 4 FTC, 4 MTC and 1 primitive thyroid paraganglioma); 32/53 (60.4%) had microPTCs	14/53 (26.4%) pts had multifocal tumours and in 12/53 (22.6%) were bilateral
Negro and cols., 2013*	2000 - 2010	Italy	970 pts	TT	84/ 970 (8.7%) pts had TC	
Botrugno and cols., 2011	2000 - 2008	Italy	462 pts	TT	41/462 (8.9%) pts had TC; the most common histotype was PTC	
Gelmini and cols., 2010	10 yrs	Italy	739 pts	TT in 503/739, PT in 239/739	82/739 (11.1%) pts had TC, mainly microPTC	Lymph-node metastases were found in the 3.6% of cases
Pisello and cols., 2007	Jan 2000 - Jan 2006	Italy	502 pts	TT in 458/502, PT in 44/502	17/502 (3.4%) pts had microPTC	In 34/502 (6.8%) pts, tumours were suspected preoperatively; 2/502 (0.4% had multifocal microPTCs
Carlini and cols., 2006*	n/a	Italy	88 pts	TT	19/88 (21.6%) pts had TC	
Miccoli and cols., 2006	Feb 2002 - Nov 2003	Italy	998 pts	TT in 902/998 pts, PT in 96/998	104/998 (10.4%) pts had TC; the most common histotype was PTC (99/104)	Tumours were multifocal in 19.8% of the cases
Carlini and cols., 2005*	1-year	Italy	n/a	n/a	Incidence of microTC was 27.4%	
Pingitore and cols., 1993	1985 - 1991	Italy	2930 pts	n/a	132/2463 (5.4%) pts had TC	2463/2930 pts were considered clinically benign and 467/2930 pts malignant, preoperatively
Pascual Corrales and cols., 2012	n/a	Spain	372 pts	n/a	58/372 (15.6%) pts had TC	Pts underwent surgery for: 49 pts (EMNG), 8 pts (GD) and 1 pt (HMNG)
Slijepcevic and cols 2015	., 2008-2013	Serbia	2466 pts	TT or PT	403/2466 (16.3%) pts had microPTC	
Zivaljević and cols., 2008	2004	Serbia	578 pts	n/a	53/578 (9.2%) pts had microTC	Pts underwent surgery for: 201 pts (MNG), 178 pts (thyroid adenoma), 89 pts (GD), 79 pts (PD) and 31 pt thyroiditis
Alecu and cols., 2014	2002 - 2012	Romania	145 pts	TT in 102/145 pts, PT in 43/145	10/145 (6.9%) pts had microPTC	
Muntean and cols., 2013	2002 - 2011	Romania	2168 pts	TT or PT	187/2168 (8.6%) pts had microPTC	In 66/187 (35.3%) pts had multifocal tumours, and in 31/187 (16.6%) were bilateral
Lukás and cols., 2010	2004 - 2008	Czech Republic	400 pts	TT or PT	34/400 (8.5%) pts had microTC; 32/34 (94.1%) were microPTCs	In 5/34 (14.7%) pts had multifocal tumours and in 4/34 (11.8%) were bilateral
Nechaĭ and cols., 2012*	2008 - 2009	Ukraine	608 pts	n/a	56/608 (9.2%) pts had TC; 43/56 (76.8%) were microPTCs	
Barczyński and cols., 2011*	1999 - 2009	Poland	8132 pts	TT in 2918/8132 pts, PT in 5214/8132	406/8132 (5.0%) pts had TC	
Vasileiadis and cols, 2013	2001 - 2009	Greece	2236 pts	TT	268/2236 (12.0%) pts had microPTC.	
Siassakos and cols. 2008	Jan 1997 - Jul 2001	Greece	191 pts	TT	29/191 (15.2%) pts had microTC (18 microFTC, 10 microPTC and 1 microMTC)	In 8/29 (27.6%) pts had multifocal microcarcinomas
Sakorafas and cols. 2007	Feb 1990 - Feb 2002	Greece	380 pts	TT in 377/380 pts, PT in 3/380	27/380 (7.1%) pts had microPTC	In 11/27 (40.7%) pts had multifocal tumours
Yazici and cols., 2015	2010-2013	Turkey	86 pts	TT or PT	6/86 (7.0%) pts had TC (4 microPTC and 2 PTC)	
Tasova and cols., 2013	Mar 2007 - May 2011	Turkey	443 pts	TT in 401/443, PT in 42/443	66/443 (14.9%) pts had TC (56 PTC, 4 FTC and 6 indeterminate lesions)	
Karakoyun and cols 2013	Jan 2010 - Aug 2011	Turkey	50 pts	TT	5/50 (10.0%) pts had microPTC.	
Berker and cols., 2011	Jan 2004 - Jan 2009	Turkey	337 pts	TT	18/337 (5.3%) pts had microTC	Pts underwent surgery for: 278 pts (MNG), 59 pts (GD)
Tezelman and cols., 2009	1988-2007	Turkey	2906 pts	PT in 1695/2906 (group 1), TT in 1211/2906 (group 2)	210/2906 (7.2%) pts had TC (81 in group 1 and 129 in group 2)	
Giles and cols., 2004	Sep 2001 - Dec 2002	Turkey	218 pts	TT in 109/218 (group 1), and PT in 109/218 (group 2)	18/218 (8.3%) pts had PTC (10 in group 1 and 8 in group 2)	All pts underwent surgery for MNG
Fernando and cols., 2009	2003 - 2005	Sri Lanka	68 pts	TT	6/68 (8.8%) pts had TC (2 PTC, 2 MTC and 2 FTC)	
John and cols., 2014	Jan 2005 - Jun 2012	India	1300 pts	TT or PT	94/1300 (7.2%) pts had microPTC	
Wu and cols., 1993	1962 - 1991	China	135 pts	n/a	54/135 (40.0%) pts had TC	
Koh and cols., 1992	n/a	Malaysia	107 pts	n/a	8/107 (7.5%) pts had TC, mainly PTC	All pts underwent surgery for MNG
Preece and cols., 2014	Sep 1994 - Aug 2012	Australia	1508 pts	TT	96/1508 (6.4%) pts had TC	Pts underwent surgery for: 963 pts (MNG), 295 pts (TNG), 250 pts (GD)
Bron and cols., 2004	1998-2002	Australia	834 pts	TT	71/834 (8.5%) pts had TC (33 microPTC, 22 PTC, 11 FTC, 5 other)	74/834 had previously undergone PT
Bhuiyan and cols., 2015	2003-2008	South Africa	90 pts	TT	10/90 (11.1%) pts had TC (3 PTC and 7 FTC)	
Bombil and cols., 2014	2005-2010	South Africa	162 pts	TT	4/162 (2.5%) pts had PTC (3/4 were FV-PTC)	
Edino and cols., 2010	2000-2006	Nigeria	160 pts	n/a	24/160 (15.0%) pts had TC (13 FTC, 10 PTC, 1 MTC and 1 ATC)	In 6/25 (24.0%) pts tumours were detected preoperatively by FNAC
Choong and cols., 2015	1990-2014	USA	148 pts	TT (120 pts) or PT (28 pts)	7/148 (4.7%) pts had TC (5 PTC, 1 FTC and 1 MTC)	All pts underwent surgery for TNG
Ergin and cols., 2014	2005-2013	USA	493 pts	TT	69/248 (28%) pts in EG group and 64/245 (26%) pts in GD group had microPTC	Pts underwent surgery for: 248 pts (EG), 245 pts (GD)
Bahl and cols., 2014*	2003-2012	USA	2090 pts	n/a	680/2090 (33%) pts had TC	
Phitayakorn and cols., 2013	Dec 1985 - Mar 2010	USA	300 pts	TT or PT	31/300 (10.3%) pts had TC (22 microPTC, 8 PTC and 1 FTC)	
Phitayakorn and cols., 2008	1990-2007	USA	506 pts	TT, PT in 10 pts with GD	11/333 (3.3%) nonTNG pts had PTC, 2/92 (2.2%) GD pts had microPTC, 5/81 (6.2%) TNG pts had TC (3 PTC, 1 FTC and 1 MTC)	Pts underwent surgery for: 333 pts (nonTNG), 92 pts (GD), 81 pts (TNG)
Smith and cols., 2013	2000-2011	USA	1523 pts	n/a	238/1523 (15.6%) pts had TC (175 PTC, 39 FV-PTC, 11 FTC and 13 other malignancies)	
Smith and cols., 2013*	2002-2011	USA	164 pts	n/a	30/164 (18.3%) pts had TC	All pts underwent surgery for TNG
Dunki-Jacobs and cols., 2012	2001-2007	USA	723 pts	TT or PT	194/723 (27%) pts had TC (PTC or microPTC)	In 137/194 (70.6%) pts, tumours were suspected preoperatively
Bradly and cols., 2009	Jan 2000 - May 2008	USA	678 pts	TT or PT	81/678 (12%) pts had PTC	
Lokey and cols., 2005*	Dec 1998-Dec 2003	USA	738 pts	n/a	28/738 (3.8%) pts had TC (mainly microPTC)	

pts: patients; TT: total thyroidectomy; PT: partial thyroidectomy; TC: thyroid carcinoma; PTC: papillary thyroid carcinoma; FV-PTC: follicular variant of papillary thyroid carcinoma; FTC: follicular thyroid carcinoma; MTC: medullary thyroid carcinoma; ATC: anaplastic thyroid carcinoma; MNG: multinodular goiter; UNG: uninodular goiter; TNG, toxic nodular goiter; GD, Graves’ disease; PD, Plummer's disease; HT, Hashimoto's thyroiditis; EG, euthyroid goiter; EMNG, euthyroid multinodular goiter; HMNG, hyperthyroid multinodular goiter, FNAC: fine-needle aspiration cytology; n/a: not available.

* Tabulated data taken from the abstracts written in English and/or illustrative material.

## DISCUSSION

The increased incidence of thyroid carcinoma seems to be related to an improved diagnostic approach, given by a widespread use of US and cytology, but also by the employment of new imaging techniques, such as ^18^F-fluoro-deoxyglucose positron emission tomogram/computed tomography (^18^F-FDG-PET/CT) (69–71). Among patients who performed neck US for suspected parathyroid disease, incidental thyroid nodules were found in 46% of them (72). Similarly, thyroid incidentalomas discovered during CT or magnetic resonance imaging that had been carried out for other reasons have been reported with an incidence of 16% (73,74); moreover, 9% to 13% were discovered during carotid US (75,76), and 2% to 3% at ^18^F-FDG-PET/CT scan (77–79). The prevalence of incidental thyroid nodules on US in the general population ranges between 42% and 67% (80,81). In thyroidectomy specimens, ITC prevalence ranges up to 40% (2). In autopsy studies, the reported prevalence of ITC ranges from 0.01% in USA to 35.6% in Finland (7).

The overview of the literature (Table 3, refs. 23-68), has shown that one-third (n = 16) of the studies are on cohorts of thyroidectomized patients smaller than ours (n = 50 to 191, compared to 207), and oneseventh of the studies (n = 7) are on cohorts slightly greater than ours (256 to 8,132). Prevalence of ITC at surgery ranges between 2% and 40% (1,2,17,23–68). In Europe, the frequency of ITC varies from 2.2% to 27.4%, and a similar wide range (3.3% to 33%) is observed in the United States. Interestingly, in Eastern Europe (Romania, Czech Republic, Ukraine, Poland), the frequency of thyroid cancer is relatively low (range 5-9.2%). In Turkey, excluding the study from Tasova and cols. (46), there is a lower variation range in the reported incidence of thyroid cancer (7-10%).

One comment deserves the coexistence of ITCs with HT. We found a 33% rate of ITCs (always microPTCs) in patients with histologically confirmed HT. This rate is greater than that reported in one recent retrospective study from Serbia (37). Slijepcevic and cols. (37) also investigated the prevalence of microPTC in patients operated for benign thyroid diseases in a retrospective study of 2,466 patients who underwent thyroid surgery from 2008 to 2013. The overall prevalence of microPTC was 16.3%, the highest being in HT. Smith and cols. (63) examined cancer frequency in patients referred for removal of benign thyroid disease in a multiinstitutional series of 2,551 patients. Indeterminate/malignant FNA diagnoses were excluded (n = 1,028). Overall, 238 (15.6%) cancers were found, and 275 patients had thyroiditis (18%). Presence of thyroiditis was not associated with cancer, because there were 47 ITCs in the 275 patients compared with 191 ITCs in 1,247 patients without thyroiditis (17.1% *vs* 15.3%). Our rate of 33.3% was highly significant as well as the 22.7% (χ^2^ = 10.80, P < 0.001) of Slijepcevic and cols. (37), whereas the rate of 17.1% (χ^2^ = 0.388, P = 0.533) reported by Smith and cols. did not reach statistical significance.

The limitations of this study are due to its retrospective nature. Another limitation is the natural history of thyroid cancer, which is a slow growing tumor, so that extended follow-up is needed to evaluate the long-term outcomes. The strength of the study lies in its short course, avoiding that a variable number of pathologists histologically examined the specimens using different methods of evaluation.

Our 12.1% rate is comparable to rates from other European countries, including Belgium, Italy (25,30,33) and Greece (17). Because Italy and Greece have no nuclear plants, we tend to exclude that our rate was influenced by the relative vicinity of our medical center and residence of patients to three nuclear plant units (82). A systematic review and meta-analysis on this issue does not support an association between living near nuclear power plants and risk of thyroid cancer. However, sensitivity analysis by exposure definition demonstrated that living less than 20 km from nuclear power plants was associated with a significant increase in the risk of thyroid cancer (83). Additionally, with a 12% risk that MNG harbors preoperatively unsuspected PTCs which can have already infiltrated the capsule and that are accompanied frequently by other PTC foci contralaterally, an adequate surgical approach has to be considered.

The operative management of benign thyroid diseases includes partial and total thyroidectomy: the first one preserves thyroid function, sparing patients the need for lifelong thyroid hormone replacement (84); moreover, microPTCs can have an excellent prognosis not requiring completion thyroidectomy. On the other hand, total thyroidectomy may present complications, such as hypoparathyroidism (often transient) (85) and recurrent laryngeal nerve injury (84), which occurs in 6% and 1% of patients, respectively (84). However, reoperation after partial thyroidectomy can be needed in cases with multifocal thyroid cancer or for radioactive iodine ablation.

In our experience, total thyroidectomy showed neither mortality nor transient and permanent nerve injuries, avoiding the risk of recurrence and necessity of completion thyroidectomy, with its known technical difficulties and increased risk of complications, and also avoiding the risk of ITC presence in remnant tissue.
